# The Policy of Expansion

**Published:** 1911-08

**Authors:** 


### BUFFALO MEDICAL JOURNAL

**A Monthly Review of Medicine and Surgery**

*EDITOR*
WILLIAM WARREN POTTER, M. D.

All communications, whether of a literary or business nature, books for review and exchanges, should be addressed to the editor, 238 DELAWARE AVENUE, BUFFALO, N. Y

VOL. LXVII. AUGUST, 1911. No. 1

### The Policy of Expansion

Beginning with the sixty-seventh year, the BUFFALO MEDICAL JOURNAL will adopt a policy of expansion. We wish to increase the thickness of the magazine, while condensing its contents and increasing their practical value, to carry more advertisements, to be less local. This policy requires some time and much labor, both on the part of the staff of the Journal, and of the physicians of Buffalo and Western New York.

We want the medical profession to feel that they own the Journal—No, there is no cause for alarm, there will be no importuning for purchase of stock. Nor does this kind of ownership imply the feasibility of insistance on the carrying out of individual opinions, much less of combating individual antipathies. But we want every physician in Western New York to contribute to making the highest ideal a realisation, to use the Journal as a medium of communicating to the profession, his best thoughts and knowledge of his personal movements and interests.

The "circulus vitiosus" is well understood by medical men. It may affect a periodical as well as a patient. It would be better if we thought more of the "circulus favorabilis." So far as a periodical is concerned, this means more subscribers, more advertisers, more funds, more pages, better quality, and so on, over and over. In looking over the logical field of the Journal, we note that, outside of New York City, there is no medical journal published in the state, except the *Albany Medical Annals,* which is the official organ of the Albany Medical College. While the BUFFALO MEDICAL JOURNAL reaches adjoining states and Ontario, and has a surprisingly large circulation of wide radius, even going across the Atlantic, both as an exchange and to subscribers, we feel that it should represent especially, the interests of the four western District Branches of the Medical Society of the State of New York or, rather of their component societies, independent organisations and individual members. This region has a population of about 2,500,000, a professional strength of about 4,000, of which about half is included in regular medical organisations, state, district and county. We feel that, while maintaining the American precedents of individual freedom of thought and action and of local self-government, the medical profession should concentrate its forces by thorough organisation and, in both of these balanced and contrasted but not opposed policies, the BUFFALO MEDICAL JOURNAL expects to be of greater and greater service.

A story is told of a retired army officer who met a brother officer, less fortunate, selling pies on a street corner. He began a voluble expression of sympathy but was cut short with "Hang your sympathy, buy a pie." The BUFFALO MEDICAL JOURNAL is not in need of sympathy but, nevertheless, to carry out the aim mentioned, it needs an equally practical spirit of co-operation. This means, prompt payment of subscriptions, personal effort to secure new subscribers, on the part of those already on our list, remittance of draft, post office order or stamps on the part of those who read this and who are not already subscribers. It should not be forgotten that an advertisment implies, not merely a desire to sell wares, but a very practical assistance in the educational work of periodicals, and, in particular, a disposition to cordial co-operation with the profession.

There is one detail which we have only recently realised and which, we believe, is not commonly appreciated. Owing to family relationship, association in institutions and partnership, at least in sharing offices, a circulation which covers the entire medical profession of any average city has a numeric—and financial—strength of not much more than half of the medical population. In the last three days, spontaneously and in response to telephonic requests, thirty-five additions have been made to our list. This brings us nearly to the possible limit for Buffalo and we ask the co-operation of our readers in bringing in the comparatively few remaining.

It would involve a very considerable expense to solicit, even by sample copy and circular postal card, subscriptions from the other cities and smaller communities of Western New York, following a directory list. We prefer to invest this sum more directly in enlarging the JOURNAL. We are, therefore, mailing to each post office in the territory indicated, one or a few copies of this issue, according to population, and ask, as a special favor, that the physician addressed will donate a little time at the telephone, or personally, in calling the attention of his confreres to this Journal. No formalities will be necessary in the way of order blank, and the like. Simply send draft, order, check or a sheet of postage stamps to the amount of $2.00.

